# Serologic evidence of seasonal influenza A and B in HIV patients on combined antiretroviral therapy in Lagos, Nigeria

**DOI:** 10.4102/ajlm.v9i1.1048

**Published:** 2020-12-21

**Authors:** AbdulAzeez A. Anjorin, Barakat A. Adepoju

**Affiliations:** 1 Department of Microbiology, Faculty of Science, Lagos State University, Ojo, Nigeria

**Keywords:** influenza A, influenza B, HIV co-infection, seroprevalence, Lagos

## Abstract

**Background:**

Influenza and HIV are endemic in Nigeria but there is no epidemiological data on the co-infection of influenza A and B among HIV patients.

**Objective:**

We investigated seasonal influenza A and B, and co-infection among HIV patients on combined antiretroviral therapy (cART) in Lagos, Nigeria.

**Methods:**

In a prospective cross-sectional study, clear sera collected from 174 HIV-positive patients between August and September 2018 were analysed for immunoglobulin M-specific antibodies to seasonal influenza A subtypes H1N1 and H3N2, and influenza B by enzyme immunoassay.

**Results:**

A total of 39.7% (69/174) of HIV patients were seropositive for influenza A or B viruses with 84.1% (58/69) being positive for influenza A, 13.04% (9/69) seropositive for both influenza A and B, and only 2.9% (2/69) positive for influenza B mono-infection. Median age was 44 (mean 45, mode 40, range 18–74) years. The 41–50 years age group had the highest seroprevalence (39.1%; 27/69). Seropositivity was highest among women (65.2%; 45/69). A total of 88.4% (61/69) of HIV patients seropositive for influenza A or B were on fixed dose cART, while 73.9% (51/69) were virologically suppressed. Furthermore, 27.5% (19/69) were immunocompromised, of which 21.1% (4/19) were severely immunosuppressed (cluster of differentiation 4 < 200 cells/mm^>3^).

**Conclusion:**

Influenza A and B was prevalent among HIV patients on cART, which may predispose them to life-threatening complications. We recommend strong advocacy on the need to reduce the risk of exposure to influenza and for the provision of an influenza vaccine in Nigeria.

## Introduction

In humans, seasonal influenza viruses cause an annual 3–5 million illnesses and 290 000–650 000 deaths worldwide.^1^ The majority of the deaths and severe illness occur in low- and middle-income countries^2^ especially in Africa where there is little or scanty information on epidemiological surveillance. Influenza virus infection can be self-limiting and could vary in severity from asymptomatic to fatal disease arising from medical complications. These complications include pneumonia, meningitis and worsening of underlying medical conditions, mostly in immunocompromised individuals.^1^

On the other hand, HIV is a retrovirus that spreads through the body rapidly, causing damage to the immune system by attacking host cluster of differentiation 4 (CD4) cells with resultant AIDS.^3^ In 2017, HIV-related causes killed about 1 million people worldwide, having claimed over 35 million lives in the last three decades.^4^

According to the World Health Organization,^4^ as of 2018, 1.8 million newly infected individuals were added to the 36.9 million people living with HIV, with 25.7 million (> 66%) infected people living in the sub-Saharan Africa region.^4,5^ There are about 197 997 230 people living in Nigeria, equivalent to 2.57% of the total world population (www.worldometers.info), with up to 3.2 million people living with HIV.^6^ This is the second-largest HIV epidemic after South Africa and represents 9% of the total population living with HIV globally, among whom 1 million people are on antiretroviral therapy (ART).^6,7,8^ Lagos State with a total population of about 21 million has a HIV prevalence of 2.2%.

Influenza viral infection is a common cause of respiratory illness among HIV-infected persons in whom it can be more severe and prolonged.^9^ HIV has also been described as the most common underlying risk factor in patients with respiratory infection.^10^ Influenza virus co-infections in HIV-infected patients cause a 4–8 times higher incidence of hospitalisation and death compared to non-HIV-infected individuals.^11^ The reduction in the number of CD4 T-cells due to infection with HIV causes immunodeficiency and thus higher susceptibility to complications of influenza in HIV patients.^12^

In general, the immunogenicity of influenza viral infections in HIV-infected patients is directly proportional to the CD4 cell count and correlates negatively to the viral load.^13^ Studies have also shown that better immune health (i.e. higher CD4 cell counts and suppressed viral load of HIV patients on combined antiretroviral therapy [cART]) in comparison with immunocompromised patients made them less likely to experience prolonged shedding of influenza virus.^9^

However, a lack of epidemiological data on seasonal influenza in HIV-positive individuals is a major problem preventing the development of national policies on influenza preventive strategies in sub-Saharan Africa, including Nigeria. In South Africa, 44% of influenza positive patients having acute respiratory infections are HIV-infected.^10^ No data is available on influenza virus infection in HIV patients from Nigeria and that underscores the importance of this study.

Serological assays usually complement epidemiological and clinical investigations in the detection and identification of influenza viruses. They measure antibodies developed in response to infection with antigenically novel and seasonal influenza viruses.^14^ The enzyme-linked immunosorbent assay method has been used in different studies for detecting serological evidence of the influenza virus in humans^15,16,17^ because it is apt, easy, fast, can be automated and is commercially available for large-scale sero survey, targeting specific antibodies against different subtypes of influenza virus. It is also affordable for sero epidemiology in poor and limited resource settings.

This study was therefore designed to investigate seasonal influenza A and B virus infections in HIV patients on cART in Lagos, Nigeria.

## Methods

### Ethical considerations

Permission was sought from the head of the AIDS prevention initiative in Nigeria (APIN) clinic where the samples were collected after tendering the ethical approval (reference no.: LREC.06/10/1030) obtained from the Health Research and Ethics Committee of the Lagos State University Teaching Hospital (LASUTH).

### Study area, population and design

A prospective cross-sectional study was designed. Outpatients attending the AIDS prevention initiative in Nigeria (APIN)-Lagos University Teaching Hospital clinic who were previously laboratory confirmed to be HIV-positive were recruited for this study from August 2018 to September 2018. APIN-Lagos University Teaching Hospital clinic is a large university-based HIV clinic in Lagos, Nigeria, with about 15 000 patients currently enrolled in Lagos. After explaining the concept of the study, a total of 174 HIV patients, including 165 on cART, who were unvaccinated for influenza and gave both oral and written informed consent, were bled by venepuncture. Demographic and clinical data were appropriately collected with a designed questionnaire. Results of further laboratory analyses including CD4 count, RNA viral load and cART regimen were obtained from the patients’ clinical records.

### Sample collection and treatment

Approximately 5 mL of whole blood samples were collected into sterile plain bottles. They were stored in sample coolers stacked with ice packs before being conveyed to the laboratory. The samples were centrifuged at 704 × g for 15 min to obtain clear sera that were aliquoted into labelled sterile plain cryovial tubes. The sera were then stored away at −30 °C until ready for serological analysis.

### Laboratory analysis

Clear sera were analysed for the detection and quantitative determination of immunoglobulin M antibody specific for influenza virus by enzyme-linked immunoassay (Demeditec Diagnostics GmbH, Kiel, Germany) in the Department of Microbiology (Virology Research) Laboratory, Lagos State University, Ojo. Following manufacturer’s instructions, sufficient amounts of micro titre wells were prepared for the standards, controls and samples as well as for the substrate blank. The samples were diluted with ready-to-use sample diluent provided with the test kit in the ratio 1:100 (2 *µ*L serum + 200 *µ*L sample diluents). Assay absorbance was read at a wavelength of 450 nanometres with an EMax precision Microplate reader (Molecular Devices, LLC, San Jose, California, United States). Sample results were compared with the included standards and controls and were interpreted based on the assay standard curve as recommended by the manufacturer. It should however be noted that positive immunoglobulin M antibody specific for influenza virus implies a recent immunological reaction to circulating live influenza strains as a result of infection since Nigeria does not currently practise influenza vaccination.

### Statistical analysis

Raw data were entered into Microsoft Excel, version 2013 (Microsoft Corporation, Redmond, Washington, United States). Descriptive statistics was performed while inferential statistics was analysed with chi-square at *p* less than 0.05 for statistical significance using GraphPad Prism version 8.0.1 (GraphPad Software Inc., San Diego, California, United States).

## Results

Out of the 174 HIV-positive patients tested, 69/174 (39.7%) were seropositive for influenza A or B viruses, with 58/69 (84.1%) positive for influenza A, 2/69 (2.9%) for influenza B, and 9/69 (13.0%) for both influenza A and B (Table 1). The median age of patients was 44, mean 45, mode 40, and range 18–74 years. Seropositivity was higher in female patients (45/69; 65.2%) compared to male patients (17/69; 24.6%). A total of 51/69 (73.9%) of the patients were virologically suppressed with HIV RNA under 400 copies/mL, and 19/69 (27.5%) were immunocompromised (CD4 < 400 cells/mm^3^). Out of the immunocompromised patients, 4/19 (21.1%) were severely immunosuppressed (CD4 < 200 cells/mm^3^). 61/69 (88.4%) of HIV patients seropositive for influenza A or B were on fixed dose cART compared to those that were seronegative: 96/105 (91.4%) (*p* < 0.001).

**TABLE 1:** Characteristics of HIV patients positive for influenza A and B viruses in 2018 in a university-based HIV clinic in Lagos, Nigeria.

Characteristics	No. of patients (*n* = 174)	Total influenza positive (*n* = 69)		Influenza A positive (*n* = 58)		Influenza B positive (*n* = 2)		Co-infection of influenza A and B (*n* = 9)		*p*
		*n*	%	*n*	%	*n*	%	*n*	%	
**Gender**										
Female	112	45	65.2	38	65.5	2	100	5	55.6	0.61
Male	55	17	24.6	15	25.9	0	0	2	22.2	
Unknown	7	7	10.1	5	8.6	0	0	2	22.2	
**Others**										
HIV RNA < 400 copies/mL	144	51	73.9	42	72.4	2	100	7	77.8	< 0.001
CD4 count < 400 cells/mm^3^	59	19	27.5	15	26.0	1	50	3	33.3	< 0.001
Combined antiretroviral therapy	165	61	88.4	52	89.7	2	100	7	77.8	< 0.001

Note: *p*-values were obtained by comparing the seropositive and sero-negative variables of influenza A, B and co-infection foreach characteristic understudied.

CD4, cluster of differentiation 4; RNA, ribonucleic acid; HIV, human immunodeficiency virus.

The most commonly prescribed cART used as a single-pill combination at the APIN clinic included atazanavir, azidothymidine, efavirenz, lamivudine, lopinavir or ritonavir, nevirapine, and tenofovir (Table 2). The majority of patients received a combined therapy of tenofovir, lamivudine and efavirenz (74/174; 42.5%) or azidothymidine, lamivudine and nevirapine (64/174; 36.8%).

**TABLE 2:** Commonly prescribed combined antiretroviral therapy regimens in HIV patients positive for influenza virus immunoglobulin M antibodies in 2018 in a university-based HIV clinic in Lagos, Nigeria.

cART regimen	No. of patients	Total influenza positive		Influenza A positive		Influenza B positive		Co-infection of influenza A and B	
		*n*	%	*n*	%	*n*	%	*n*	%
FDC (TDF/3TC/EFV)	74	28	37.8	23	82	2	7.1	3	10.7
FDC (AZT/3TC/NVP)	64	20	31.3	19	95	0	0.0	1	5.0
FDC (TDF/3TC)-AZT-LPV/r	4	2	50.0	2	100.0	0	0.0	0	0.0
FDC (TDF/3TC)-LPV/r	4	3	75.0	2	66.7	0	0.0	1	33.3
FDC (TDF/3TC)-AZT-ATV/r	3	2	66.7	1	50.0	0	0.0	1	50.0

cART, combined antiretroviral therapy; FDC, fixed dose combination; TDF, tenofovir; 3TC, Lamivudine; EFV, efavirenz; AZT, azidothymidine; NVP, nevirapine; LPV, lopinavir/ritonavir (r); ATV, atazanavir;

HIV, human immunodeficiency virus.

**FIGURE 1:**
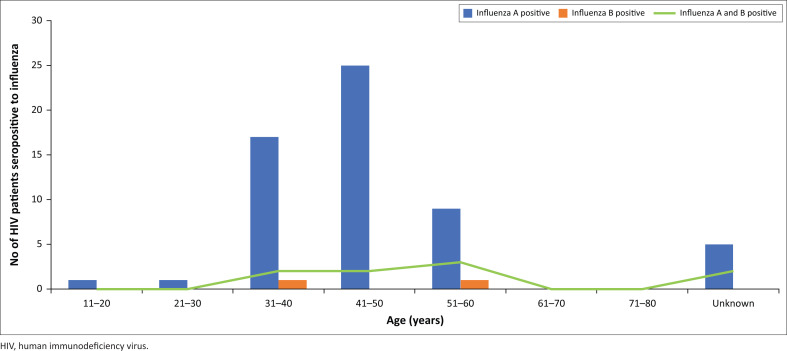
Age (years) distribution of HIV patients seropositive to influenza in 2018 in a university-based HIV clinic in Lagos, Nigeria.

Seroprevalence was highest in patients aged 41–50 years (39.1%; 27/69), with no detections in the age groups 61–70 and 71–80 years (Figure 1).

## Discussion

In this study, we described the recent infection of seasonal influenza A and B, and their co-infection, among HIV patients on cART in Lagos, Nigeria, with a high predominance of influenza A virus. While a high proportion of the patients were virologically suppressed, having HIV RNA under 400 copies/mL, 27.5% of them were immunocompromised while 21.1% were severely immunosuppressed (CD4 < 200 cells/mm^3^). The hospital population-based study revealed an overall seasonal influenza serological prevalence of 39.7% (69/174) that will serve as a baseline for other studies in Nigeria. We defined the seasonal influenza virus as any type or subtype of influenza virus including influenza A or B that are commonly circulating in our environment at any particular point in time as against a pandemic influenza virus. To our knowledge, this is the first study on clinical and sero-epidemiological prevalence of influenza viruses in HIV patients on cART in Nigeria.

The seroprevalence rate is comparable with the 31% serological response recorded in HIV-positive individuals in Miami, United States.^18^ A significantly lower seroprevalence rate of 14.7% was recorded in Taiwan.^19^ A higher prevalence rate of 71.6% for influenza-specific antibody responses in HIV patients was recorded in Rome, Italy.^20^ In Africa, lower prevalence rates of 11% and 24.5% were also recorded in Malawi and Kenya.^21,22^ The disparity observed in the prevalence rates could be attributed to differences in many factors including sample size and the assay type used.

Our investigation further revealed that 84.1% (58/69) were immunoglobulin M seropositive for influenza A, indicating an epidemiological predominance of the influenza A over the influenza B virus during the study period in Lagos, Nigeria. In a study conducted in Malawi,^21^ it was also reported that in low-resource settings with high HIV prevalence, there is a high prevalence of influenza illness and greater risk of hospitalisation among HIV-infected persons which supports the high prevalence rate of influenza A detected in this study.

Interestingly, 13.04% (9/69) of the HIV patients were seropositive for both influenza A and B viruses. Co-infection of influenza A and B viruses in HIV patients may exacerbate the immunocompromised status by further impairing T-cell responses with resultant fatal consequences in such individuals. This position agrees with previous studies that influenza in HIV-infected persons leads to increased and prolonged hospitalisation, and an elevated risk of in-hospital death.^9,11^ Furthermore, it is evident from previous reports that even mono-infection due to influenza A (H1N1) pandemic09 (now seasonal influenza virus) caused severe pneumonia leading to acute respiratory distress syndrome and multiple organ dysfunction associated with deaths.^23,24^ Hence, simultaneous detection of influenza A and B in patients calls for serious public health intervention to ameliorate the menace of influenza co-infection especially in people living with HIV.

Our study demonstrated the highest 39.1% seroprevalence of influenza among HIV patients in the age group 41–50 years which disagrees with the United States Centers for Disease Control and Prevention report that persons older than 65 years are more likely to be at risk of the influenza virus.^25^ Our observation is however supported by earlier findings in South Africa in 2009 to 2011^11^ and the United States in 2013^26^ which reported that on account of hospitalisation and mortality rates, older adults do not have the highest rate of infection and do not represent the main contributors to local outbreaks.

Based on gender, a higher 65.2% seroprevalence was detected among women compared to 24.6% among men in our study, which is consistent with findings from Malawi^27^ that reported higher seroprevalence among women. In contrast, a United States study recorded a higher seroprevalence in men than in women.^9^ A suggested reason for the higher seroprevalence among women could be the overwhelming burden of HIV that affects more women in sub-Saharan Africa making them more susceptible to influenza infection.

Of the 19/69 (27.5%) immunocompromised patients seropositive for the influenza virus, 4/19 (21.1%) were severely immunosuppressed (CD4 < 200 cells/mm^3^). However, the role of CD4 and its impact on influenza in HIV-positive patients is poorly understood, partly due to their heterogeneity and lack of epitope-specific systems.^28^

Contrary to studies in South Africa from 2009 to 2011^11^ and 2012–2016,^29^ the prevalence of influenza in the current study was higher among individuals with a CD4 count over 400 compared to those with a lower CD4 count. However, it should be noted that the South African study^11^ reported influenza based on detection of virus nucleic acids in symptomatic patients whereas the current study detected with serology a fraction of patients who had asymptomatic influenza infections. Similar to this study, a study in the United States from 2010 to 2011^9^ recorded a higher prevalence of influenza in patients with a CD4 count over 200 cells/mm^3^ than those with less than 200 cells/mm^3^. One possible explanation for these observations is the traditionally accepted role of influenza-specific CD4 T-cells in providing help to B-cells for the production of high-quality antibodies.^30^ Hence, depletion of CD4 T-cells prior to influenza virus infection may be responsible for the low prevalence observed.

People with a viral load under 400 copies/mL (73.9%) had the highest seroprevalence of influenza virus. This agrees with a study in the United States between 2010 and 2011^9^ that detected influenza virus in 85% of virologically suppressed (HIV RNA < 400 copies/mL) patients. This indicates that reduced viraemia of HIV does not reduce the susceptibility of HIV patients to the influenza virus.

Among the 165 HIV patients on a fixed dose combination of some regularly prescribed cART including tenofovir, emtricitabine, azidothymidine, atazanavir, efavirenz, nevirapine, and lopinavir or ritonavir, 61 (37%) were seropositive for influenza A and B viruses. The direct relationship of cART on patients and their seropositivity to influenza is not well understood. Nonetheless, our study demonstrated that HIV patients on cART were susceptible to recent infection with influenza viruses. In support of this finding, a systematic review done on co-infections and comorbidities of influenza in Africa from 1900 to 2013^2^ estimated an increased risk of influenza-associated mortality among HIV-positive individuals even after the widespread introduction of cART. Also, a study in the United States from 2011 to 2012^18^ concluded that regardless of influenza vaccination and control of viral load with cART, HIV-infected patients are at an elevated risk of acquiring seasonal influenza infection. However, in Malawi, the introduction of highly active antiretroviral therapy has led to the reduction of influenza-related complications among HIV-infected patients.^31^

### Limitations

One of the limitations of this study is that positive samples for the influenza virus by enzyme linked immunoassay could not be further tested by polymerase chain reaction due to the cost implication. Also, serological evidence from different HIV treatment centres are necessary for representative data in Nigeria. Another limitation is that an HIV-uninfected control group was not included in the study for possible comparison of the prevalence rates of influenza. Finally, it should be noted that the study was conducted over one influenza season and therefore the impact of seasonal changes in influenza strains was not studied. Rather our study has provided sero-epidemiological data on the commonly circulating seasonal influenza virus in HIV patients.

### Conclusion

This study demonstrated using serology that HIV patients on cART are susceptible to both influenza A and B viruses. It however revealed low seroprevalence of influenza B mono-infection, and influenza A and B co-infections among HIV patients. Viral co-infections may further impair the already compromised immune system of HIV patients. Strong advocacy on the need to reduce risk of exposure to influenza, and for provision of an influenza vaccine in Nigeria, is recommended to prevent or reduce the complications of viral co-infection.
